# Psychological and Emotional Effects of Digital Technology on Digitods (14–18 Years): A Systematic Review

**DOI:** 10.3389/fpsyg.2022.938965

**Published:** 2022-07-07

**Authors:** Pierpaolo Limone, Giusi Antonia Toto

**Affiliations:** Learning Science Hub, University of Foggia, Foggia, Italy

**Keywords:** psychological and emotional implication, digital technologies (DTs), adolescents, techno addictions, review

## Abstract

**Background:**

The use of smartphones and other technologies has been increasing in digitods aged 14–18 years old. To further explain this relationship and explore the gap in research, this paper will appraise the available evidence regarding the relationship digital technology use and psychological/emotional outcomes and report on the strength of the associations observed between these variables.

**Methodology:**

To select relevant studies, five separate computerized searches of online and electronic databases were performed. These included PubMed (MEDLINE, National Library of Medicine), ScienceDirect, Cochrane, Scopus, and Web of Science to attain literature from January 2017 to April 2022. The author independently reviewed studies for eligibility as per the inclusion/exclusion criteria and extracted the data according to a priori defined criteria. Risk of bias was assessed using the Agency for Healthcare Research and Quality (AHRQ) for healthcare studies and Cochrane Risk Of Bias In Non-randomized Studies of Interventions (ROBINS-I) assessment tool.

**Results:**

Seven studies were included in this review. A positive relationship was found between excessive digital technology usage and negative psychological and emotional outcomes in digitods aged 14–18 (*p* ≤ 0.005). A statistically significant difference was found between girls and boys, with girls experiencing more negative outcomes than boys.

**Conclusions:**

As the evidence in this review is distinctive, it is imperative that further research be conducted to investigate any synergistic relationships among these variables on a larger scale in order to better advise public health initiatives to specifically target heightened digital technology usage in adolescents.

## Introduction

The youth of the twenty-first century have extraordinary access to digital and media technologies as a result of developing as a digital population. Consequently, the range of digital devices and media activities accessible to children is continuously growing. Due to these current swift revolutions in digitalization, researchers have reported that the youth of today belong to a multifaceted digital generation, with adolescents novel digital environments opposing those from previous cohorts (Livingstone and Helsper, 2007; Livingstone et al., 2018). Subsequently, investigating how recent digital changes can affect the psychological and emotional wellbeing of adolescence is critical in understanding implications for adolescence in the current digital era.

Existing research proposes that “digitods”, or children born after 2008, have divergent patterns of digital behavior compared to those born a decade earlier (Leathers et al., 2013; Holloway et al., 2015). As a result, digitods are among the first cohort to grow up in homes with access to transportable touch-screen devices with heightened computation power and mobility (Livingstone and Helsper, 2007; Kucirnova and Sakr, 2015). Additionally, this current cohort of digitods have parents who incline to be experienced digital operators and consumers themselves and who often approve “new” digital parental intervention approaches to indorse adolescents' safe media use and digital literateness (Brito et al., 2018). As a result of this, adolescents are progressively exposed to an assembly of digital technologies from a very early age (Mascheroni and Cuman, 2014), and this holds implications for potential wellbeing complications connected with disproportionate screen-time or inappropriate media use. To date, however, especially following the COVID-19 pandemic, little has been reported on the impact of current adolescent digital engagement on wellbeing compared to previous generations. Our study addresses this critical question to further understand children's lives and wellbeing in contemporary societies.

### Adolescences and Digital Technology Use

Hardell and Carlberg (2009) define adolescence as the period of time between the age of puberty and adult independence, during which the personality of adolescents dynamically advance and change. According to Hardell and Carlberg (2015), when equated with adults, adolescents typically are more open-minded, socially preoccupied, less agreeable, and less conscientious. They are also characteristically more impetuous and less proficient in constraining behavior (Gandhi et al., 2012). Furthermore, risk-taking and sensation-seeking are often documented in adolescents (Hardell and Carlberg, 2015) as a result of their wellbeing and life gratification being derived from other peers (Hardell, 2018). Research has reported that during adolescence, universal levels of gratification with life and self-esteem fluctuate, occasionally dropping to an all-time low (Söderqvist and Bergman Nutley, 2015; Neophytou et al., 2021). In association with this, the use of media has been reported to typically increase, reaching an initial peak in late adolescence (Tian et al., 2020). However, interestingly, research has shown that the life satisfaction and health status of the current generation does not seem to do better or worse as a result of increased media and technology use than previous generations'. This study was conducted to analyze the progression of numerous wellbeing-related factors among 46,817 adolescents and young adults in Europe. The evidence demonstrated that, while overall internet usage rose robustly, both life satisfaction and health problems remained stable. Nonetheless, concerns about the effects of new technology on adolescent development and their perceived susceptibility to the influence of digital technology as a supposedly vulnerable group have been widely debated.

The umbrella term “digital technology” incorporates countless devices, services, and types of use. Most adolescent digital technology use, however, presently tends to take place on mobile devices (Tian et al., 2020; Macedo et al., 2021). Offering the functions of many other media such as Instagram and Snapchat, smartphones play a crucial role in adolescent media use and are thus considered a “metamedium”. An illustrative survey conducted by Shelley et al. (2021), on teens in the US revealed that the utmost frequently used digital amenities are YouTube (85%), followed closely by Instagram (72%) and Snapchat (69%). All devices and services offer different functionalities and affordances; therefore, when on social media, adolescents can actively conversate with others, post, like, or share. Contrastingly, adolescents can also passively engage in use through simply loitering and observing the content of others. Lastly, a significant distinction amongst diverse types of use is whether technology use is social or non-social. Social use captures all kinds of active interpersonal communication, such as conversating and texting or liking photos or sharing posts, while non-social use encompasses (definite forms of) reading and playing, hearing music, or viewing videos. Comprehending digital technology use as a universal and generic behavior disregards the several forms such behavior can take. As a result, investigating the impact of digital technology use on adolescent psychology and emotions requires the awareness that digital technology usage is far from a uniform notion.

### Effect of COVID-19 Pandemic on Adolescent Technology Use

The COVID-19 pandemic has created obstacles in the lives of most people across the world following the implementation of social distancing and eventual lockdowns in many countries. Although lockdowns were evidenced to be crucial in curtailing the spreading of COVID-19 (Lilleri et al., 2020; Serra et al., 2021) an added mounting alarm is the impact of the lockdown on the behavioral, emotional, psychological, and neurological wellbeing of adolescents. The use of smartphones and other technologies during the pandemic has been increasing noticeably in not only parents, but children as well as they engage in activities such as gaming, online lessons, and passing the time. More specifically, the overuse of technology is also measured as an alarming factor to the mental health of adolescents (Drouin et al., 2020). Conferring to one particular study, there has been a notable 15% upsurge in technology in adolescents who reported using it “all the time” (Ammar et al., 2021). This rise in technology usage has been documented as a risk to the development of psychological conditions. Similarly, one study conducted by Hueso et al. (2021) found that the 16.4% prevalence of smartphone use in children during the pandemic was problematic.

Despite the growing body of literature on how digital technologies impact child wellbeing, previous research has provided little evidence on recent digital trends, including data from the COVID-19 pandemic. To address this gap, this systematic review examines the psychological and emotional effects of digital technology on adolescents (14–18 years), including studies conducted during the COVID-19 pandemic.

## Methodology

This systematic review was structured according to the PRISMA 2020 Statement, which is a description of 27 items to be observed when reporting on literature and systematic reviews (Panic and Ford, 2013; Agha et al., 2016; Page et al., 2021).

The primary stage of the review encompassed scoping the literature and exploring the current subject. Following this, the review questions were set and a search strategy was articulated retrospectively. Then, literature detailing data from the studies exploring the psychological and emotional effects of digital technology on adolescents (14–18 years) was reviewed. To select these studies, computerized searches of online and electronic databases were performed on PubMed (MEDLINE, National Library of Medicine), ScienceDirect, Cochrane, Scopus, Web of Science and Schola to attain literature from January 2017 to April 2022. Initially, the search terms psychological [MeSH] OR psychological effects [MeSH] AND emotional [MeSH] OR emotional effects AND digital technology OR digital [MeSH] OR technology AND adolescence [MeSH] OR child [MeSH] were used. To cover any gray areas in the literature, a search was also performed on Google Scholar using the aforementioned terms. First, to eradicate duplicates and remove non-relevant literature, all studies from all electronic databases were screened by their titles. The abstracts and full texts of all the remaining literature were then screened for eligibility using an inclusion and exclusion criteria.

The articles were selected on the basis of three guiding ideas: “psychological effect of digital technology in children or adolescents,” “emotional effect of digital technology in children or adolescents,” “psychological and emotional effects of digital technology on adolescents,” and the “effect of digital technology on adolescents in a pandemic”. Concerning the inclusion and exclusion criteria, the articles were carefully selected from peer-reviewed English journals that aimed to describe or evaluate the dimensions and variables expressed vis-à-vis the research topic aforementioned above. Publications that did not comprise the topic of interest of this systematic review or age group were excluded, as were those publications where the full text (eligibility) was not established.

**Figure 1:**
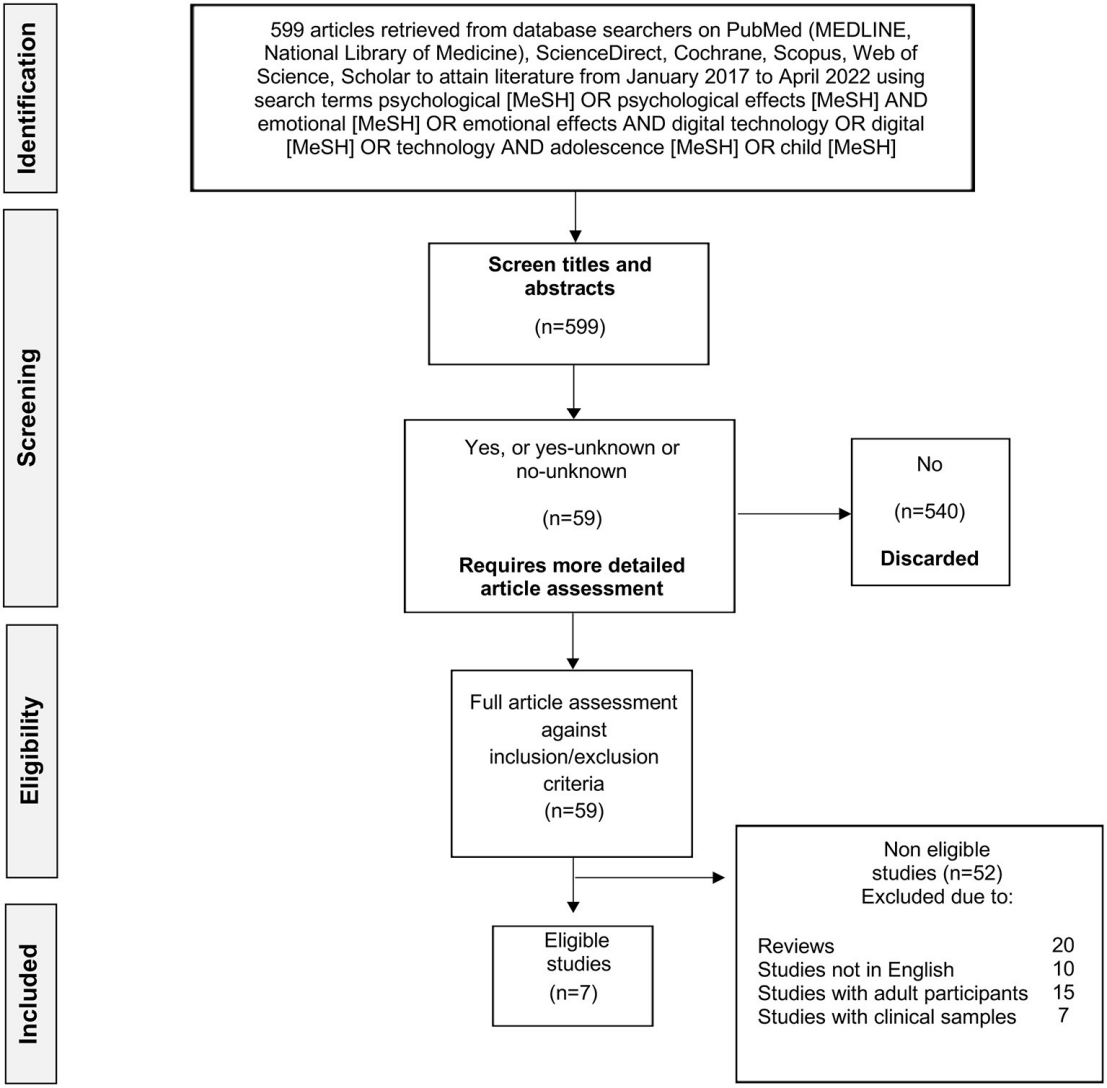
Prisma diagram of the search and selection process.

The process of including studies in the systematic review is described in Figure 1.

After the elimination of duplicates and articles in languages other than English, the search identified 599 studies consistent with the research parameters. After excluding the publications that were not relevant, seven remaining studies met the inclusion criteria.

### Inclusion Criteria

1. Studies conducted in all geographical locations, allowing for globally appropriate scientific understanding.
2. Studies conducted on Asian, Black, and Caucasian ethnicities to improve generalizability.
3. Studies conducted on child participants (between 14 and 18 years old), who constitute the population of interest.
4. Articles published between 2017 and 2022 to generate prevailing research in this area.
5. Articles in the English language for ease of scientific understanding.

### Exclusion Criteria

1. Reviews, conference abstracts, and letters to editors rather than original data to allow for fair and scientifically objective comparisons.
2. Studies where participants were diagnosed with a psychological or mental disorder to decrease risk of bias within intervention characteristics that may alter the effect of the measured outcomes.
3. Animal studies, as this is not of interest to this research topic.

### Data Extraction

The literature that passed the screening for relevance and eligibility was used for data extraction. Table 1 displays a summary of the characteristics of each study grouped by findings. Statistical significance of the effort was extracted were provided and possible. To extract the data from the literature, the following enciphering procedure was followed: (1) author/authors and year of publication, (2) title of the literature, (3) place or country of publication, and (4) key ideas of the research.

**Table 1:** Search strategy.

**Database**	**Search terms**	**Population**
PubMed (MEDLINE, National Library of Medicine), ScienceDirect, Cochrane, Scopus, and Web of Science	Psychological [MeSH] OR psychological effects [MeSH] AND emotional [MeSH] OR emotional effects AND digital technology OR digital [MeSH] OR technology AND adolescence [MeSH] OR child [MeSH]	Children and adolescents: studies including children aged 14–18 years old

### Quality Assessment

Of the selected literature that passed the screening for relevance and eligibility, data were extracted on the applied inclusion and exclusion criteria as well as the justification of these criteria. To evade data entry errors, data was extracted by the author of the paper according to the defined criteria first for literature on the psychological and emotional impact of digital technology on adolescents aged 14–18 years old. Once relevant literature was identified and obtained, they were evaluated for quality using an appropriate quality grading tool. Five studies included in this review were of non-randomized-control-trial design, thus, the Cochrane Risk Of Bias In Non-randomized Studies of Interventions (ROBINS-I) assessment tool was utilized to measure the methodological quality of the five included studies. A rating of “low” reflects the lowest risk of bias, “medium” represents an immediate and potential risk of bias in one domain, and “high” indicates the presence of bias risk in one or more of the domains. This tool has been successfully used by several reviews (Farrah et al., 2019; Igelström et al., 2021) and is sourced from a reliable institute (Cochrane); therefore, it was considered effective for use in this review. Details of the tool can be found at https://www.riskofbias.info/welcome/home/current-version-of-robins-i/robins-i-template-2016.

Two studies included in this review were of survey design, thus, the Agency for Healthcare Research and Quality (AHRQ) assessment tool was used to assess the methodological quality of the three included studies. Again, a rating of “low” reflects the lowest risk of bias, “medium” represents an immediate and potential risk of bias in one domain and “high” indicates the presence of bias risk in one or more of the domains. This tool has been successfully used by numerous reviews (Dennis et al., 2015), and therefore, it was considered effective for use in this review. Details of the tool can be found at https://effectivehealthcare.ahrq.gov/sites/default/files/pdf/assessing-the-risk-of-bias-in-systematic-reviews-of-health-care-interventions-01_0.pdf.

An independent assessment of the study quality was conducted by the author of this review. Although there was high agreement, any differences were objectively concluded. The overall quality rating of all the studies can be observed in **Table 3** and will be discussed later on in this review.

## Results

The seven selected studies included in the quality assessment are summarized in Tables 2, 3.

**Table 2:** Characteristics of selected studies grouped by outcome measure for the psychological and emotional effects of digital technology on adolescents aged 14–18 years prior to and during the COVID-19 pandemic.

**References**	**Population**	**Exposure and outcome measures**	**Level of significance**	**Effect size**
Neira and Barber, 2014	A cross-sectional study conducted with a sample of 1,819 adolescents aged 13–17 in Australia	Exposure measure: digital technology use Outcome measure: social self-concept, self-esteem, depressed mood	Results from the hierarchical regression model were considered statistically significant at *p* < 0.05 and highly significant at *p* < 0.001	Effect size was stated in terms of β and *R*^2−^values
				Social self-concept *R*^2^ = 0.03 SNS frequency β = 0.21 SNS investment β = −0.05
				Self-Esteem *R*^2^ = 0.05 SNS frequency β = 0.05 SNS investment β = −0.13
				Depressed Mood *R*^2^ = 0.09 SNS frequency β = −0.05 SNS investment β = −0.26
Sanders et al., 2019	A repeated measure study conducted on 4,013 Australian children initially aged 10–11 years who were followed longitudinally for 4 years	Exposure measure: screen time Outcome measure: social and emotional functioning and temperament profile	Paired *t*-tests used to compare baseline data with data collected after 6 weeks. ANOVA performed for between-group comparisons in the changes of study parameters at baseline and at 6-weeks follow-up. Statistical significance was set at *p* < 0.05.	Effect size was stated in terms of small effect: β = 0.1; medium effect: β = 0.3; large effect: β = 0.5.
				Quadratic effects in adjusted models with covariates: total screen time and hyperactivity SDQ subscale [βLinear = 0.028 (0.013–0.043); βQuadratic = – 0.001 (0.002–0.000); turning point: 12.29 (6.44–18.14) h; zero point: 24.59 (12.90–36.28) h] social screen time and peer SDQ subscale [βLinear = – 0.096 (– 0.159–0.034); βQuadratic = 0.011 (0.003–0.019); turning point: 4.48 (3.42–5.53) h, zero point: 8.96 (6.85–11.06) h]
Jensen et al., 2019	Observational study conducted in a sample of 388 adolescents aged 10–17 years old in US	Exposure measure: digital technology screen time Outcome Measure: mental health symptoms	A multilevel model and Linear regression models were tested at a 95% level of significance	Effect size estimates are reported as Odds Ratios, Incident Risk Ratios (OR), and standardized regression coefficients (β)
				Multilevel Model for cross-sectional associations Conduct: Texts sent OR = 1.00 Tech school work OR = 1.07 Tech communication OR = 1.05 Tech entertainment OR = 1.01 Tech creating content OR = 1.09 Total screen time OR = 1.03
				Inattention/hyperactivity: Texts sent IRR = 1.00 Tech school work IRR = 1.02 Tech communication IRR = 1.00 Tech entertainment IRR = 0.99 Tech creating content IRR = 1.01 Total screen time IRR = 1.00
				Depression: Texts sent β = 0.04 Tech school work β = 0.02 Tech communication β = −0.05
				Tech entertainment β = −0.02 Tech creating content β = −0.03 Total screen time β = −0.04
				Worry: Texts sent β = −0.01 Tech school work β = 0.04 Tech communication β = −0.02 Tech entertainment β = −0.02 Tech creating content β = 0.01 Total screen time β = −0.01
				Regression Model for Longitudinal associations adjusted for T1 risk: Conduct: Phone ownership β = −0.02 Social media access β = 0.07 Social media use frequency β = 0.06
				Inattention/hyperactivity: Phone ownership β = −0.02 Social media access β = −0.02 Social media use frequency β = −0.03
				Depression: Phone ownership β = 0.03 Social media access β = 0.03 Social media use frequency β = 0.06
				Worry: Phone ownership β = 0.04 Social media access β = −0.01 Social media use frequency β = 0.02
Kim, 2017	Multicentre prospective survey study conducted in 2,099 Korean adolescents aged 12–15 years old	Exposure measure: Social Media (e frequency of online communication or networking) Outcome measure: mental health and or suicidal thoughts	A multilevel model was tested at a 95% level of significance	Effect size estimates are reported as standardized regression coefficients (β), Odds Ratios (OR), Intraclass Correlation Coefficient (ICC), Deviance (-2LL); Social media → Mental health β = −0.016; Deviance (−2LL) = −474.60 Social media → Suicidality OR = −0.016; ICC(%) = 3.6
O'Sullivan et al., 2020	A qualitative observational study conducted in adolescents aged 14–18 in Ireland	Exposure measure: digital technology and COVID-19 lockdown Outcome measure: psychology impact	Qualitative study. A thematic approach was used. Themes are emerged by common themes and subthemes and frequencies calculated	-
Vuorre et al., 2021	A longitudinal observational study conducted in 430.561 US and UK adolescents aged 10–15 years	Exposure measure: digital technology and social media usage Outcome measure: mental health problems and symptoms	Pearson's correlations and regression models tested at 95% confidence intervals. significance was set at *p* < 0.05	Model fit was measured through the Akaike information criterion (AIC) difference. Technology use and mental health AIC difference = >3
Ravens-Sieberer et al. (2021)	A survey study conducted in 1,586 adolescents aged 11–17 years in Germany	Exposure measure: digital technology and COVID-19 pandemic Outcome measure: mental health outcomes	Independent *t*-test and linear regression model was applied at 95% level of significance. Significance was set at *p* < 0.05	Effect size was stated in terms of Cohen's *f*2 During vs. before pandemic → Mental health problems *B* = 2.18; Adjusted *R*^2^ = 0.10; parent-rpeorted (Cohen's *f*2 = 0.04)

*SNS, social networking site*.

**Table 3:** Summary of the association digital technology use and psychological/emotional outcomes in adolescents pre-and post-COVID-19.

**References**	**Variable (digital technology or COVID-19)**	**Association with psychological, emotional impact on adolescents**	**Risk of bias rating**
Kim, 2017	Digital technology	**+**	Medium
Ravens-Sieberer et al., 2021	Digital technology and COVID-19 pandemic	**+**	Medium
**Cochrane risk of bias in non-randomized studies—of interventions (ROBINS-I) assessment tool**			
Neira and Barber, 2014	Digital technology	**+**	Medium
Vuorre et al., 2021	Digital technology	**+**	Medium
Sanders et al., 2019	Digital technology	**+**	Medium
Jensen et al., 2019	Digital technology	**–**	Medium
O'Sullivan et al., 2020	Digital technology and COVID-19 pandemic	**+**	Medium

*Table includes study scores for risk of bias quality criteria as per the AHRQ tool for healthcare research and the ROBINS-I tool for non-randomized clinical trial studies*.

***+** increase in variable found; x no significant association found; – decrease in variable found; Medium = Study judged to raise some quality concerns in at least one domain but not to be at high risk of bias for any domain for result*.

In five of the seven studies, the primary objective was to assess the psychological or emotional impact of digital technology use in adolescents aged 14–18 years old (Neira and Barber, 2014; Kim, 2017; Jensen et al., 2019; Sanders et al., 2019; Vuorre et al., 2021). The primary objective of two of the seven studies was to assess the impact of digital technology during the COVID-19 pandemic on the psychological and or emotional wellbeing of adolescents aged 14–18 years old (O'Sullivan et al., 2020; Ravens-Sieberer et al., 2021). Two of the studies had cross-sectional designs (Neira and Barber, 2014; Vuorre et al., 2021), which are a form of observational study whereby the investigator measures the outcome and the exposures in the study participants simultaneously. One study employed a repeated measure design (Sanders et al., 2019), which is a research design where subjects are measured two or more times on the dependent variable and, rather than using different participants for each level of treatment, the participants are given more than one treatment and are measured after each. Two studies employed a survey (Kim, 2017; Ravens-Sieberer et al., 2021), which is a systematic method to gather information from (a sample of) entities for the purposes of constructing quantitative descriptors of the attributes of the larger population. One study was observational in design (Jensen et al., 2019), which involves a research technique where participants are observed in their most natural settings. This consequently enables researchers to assess subjects in their natural setting as opposed to structured ones like research labs or focus groups. Finally, one study was qualitative observational in design (O'Sullivan et al., 2020), which allows researchers to detect developing themes or patterns of behavior that might be ignored or obscured when using alternative methods. All the studies included an objective assessment for psychological and emotional measurement and were published in the last 10 years.

The quality scores assessed according to the AHRQ outcome and analysis reporting bias framework for the survey study revealed a medium risk of bias rating scores for two studies (Kim, 2017; Ravens-Sieberer et al., 2021). This is because for all these studies, multiple eligible outcome measures were proposed; however, no information was given regarding definitions and time points within the outcome domain. This limits the strength of the body of evidence due to the potential presence of confounding factors (from limited time point information) that have been unaccounted or unadjusted statistically for. One study received a low risk of bias rating score rating as all five domains of the quality assessment tool were accounted for (Mogharnasi et al., 2019).

The ROBINS-I tool is recommended for assessing the risk of bias in non-randomized studies of interventions, including Cochrane reviews that were observational, cross-sectional, and repeated measures levels of evidence. The quality scores, according to the tool, revealed a medium risk of bias scores for two studies that were cross-sectional in design (Neira and Barber, 2014; Jensen et al., 2019; Sanders et al., 2019; O'Sullivan et al., 2020; Vuorre et al., 2021). This is because in these studies, there were some quality concerns bias in measurements of outcomes. In particular, it is likely that the outcome assessors were aware of the intervention received by the study participants. This presents predicted direction of bias, thus limiting the strength of the body of evidence.

### Gender Differences in Emotional Impact of Digital Technology Social Media Use

Two studies reported evidence of gender differences in the emotional impact of digital technology use, particularly those centered around the use of social media. Neira and Barber (2014) reported that the main effect of the presence of a social media network profile was found to be depressed mood *p* < 0.001. Depressed mood was reported to be markedly higher for adolescents who had a social network profile compared to those who did not. The foremost impact of gender was significant for depressive moods, with females having greater levels of depressed moods than males (*p* < 0.001). The researchers also reported that the interaction between social media network profiles and gender was statistically significant for depressed mood (*p* < 0.001). There was no significant difference in depressed mood between males with and without an SNS profile.

Vuorre et al. (2021) investigated changes in mental health in relation to technology use among adolescents. The researchers reported no significant findings of the estimates that were scientifically different from zero, signifying no variation over a period of time between boys and girls aged 14–18 years old.

### Emotional Impact of Digital Technology Use

One study reported a negative relationship between digital technology use and poorer emotional consequences in adolescents aged 14–18 years old. Neira and Barber (2014) reported that the use of social network sites was not a significant forecaster of self-esteem levels; however, they found that investment in social network site usage through technology was a significant negative prognosticator of self-esteem. Additionally, the researchers reported that the more adolescents that were invested in their technology use and subsequent social network site usage, the lower their self-esteem was demonstrated to be. The association between emotional problems and social media was reported to increase by Vuorre et al. (2021). However, their association with TV remained stable. Further, these researchers revealed no credible changes in the relationship between suicidal ideation and behavior with the digital use of either social media or television mediums.

### Psychological Impact of Digital Technology Use

Two studies reported a positive relationship between digital technology use and poor psychological outcomes in adolescents aged 14–18 years old. Sanders et al. (2019) reported that weaker prosocial behavior and lower perseverance and determination were aligned with increased passive digital screen time. However, it is important to note that these results were prior to the statistical adjustment for covariates, thus weakening the power of the evidence. The researchers also reported that social screen time was linearly correlated to subordinate health-related quality of life, amplified reactivity, and worse emotional wellbeing. Similarly, Ravens-Sieberer et al. (2021) reported a positive association between digital technology use and diminished psychological health by revealing a negative impact of spending more time on the computer on mental health outcomes in adolescents in the following year, specifically, the increase in suicidal thoughts among this cohort (*p* < 0.005).

However, one study reported contrasting results. Jensen et al. (2019) reported that the adolescent use of technology did not forecast later mental-health symptoms. Furthermore, it was found that worsening mental health was not reported in the days following increased digital technology usage. The evidence from this study instead reports that adolescents were at a statistically significant risk of amplified mental-health complications. It was concluded that the findings from this study did not support the account that adolescents' digital technology use was linked with elevated mental-health indications.

### Impact of COVID-19 and Digital Technology on the Psychological and Emotional Wellbeing

Two studies reported negative psychological and emotional wellbeing outcomes due to the use of digital technology during the COVID-19 pandemic among adolescents aged 14–18 years old. O'Sullivan et al. (2020) found that children and adolescents experienced adverse mental health effects, including feelings of social isolation, depression, and anxiety and increases in maladaptive behavior. Similarly, Ravens-Sieberer et al. (2021) also found that adolescents aged 11–17 years self-reported considerable psychosomatic complaints, with approximately half of the sample (*n* = 554; 53.2%) feeling irritable and a substantial proportion of this cohort reporting sleeping problems (*n* = 449; 43.3%) and low emotions and feelings (*n* = 352; 33.8%). Additionally, girls were found to be more affected than boys with regard to and feeling low (*p* < 0.002).

## Discussion

The youth of the twenty-first century have astonishing access to digital and media technologies and increased digital companies and devices as a consequence. As a result, investigating the impact of digital technology use on adolescent psychology and emotions requires the awareness that digital technology usage is far from a uniform notion. The objective of this systematic review was to investigate the psychological and emotional impact of digital technology use in adolescents aged 14–18 years. This is of utmost importance to ensure suitably and targeted public health interventions that directly address the repercussions of the relationship that these factors hold over the health status of growing adolescents. The studies were selected according to robust inclusion and exclusion criteria focused on the association between digital technology and the psychological and emotional outcomes of adolescents aged 14–18 years old.

Researchers have documented that digital technology impacts the psychological and emotional outcomes of adolescents. The evidence of this systematic review revealed that the use of digital technology, especially in excess, negatively impacts the psychological and emotional health of adolescents (*p* < 0.005). This is consistent with the aforementioned historical studies.

Researchers have also documented that there are gender differences in the impact digital technology has on the psychological and emotional outcomes of adolescents. The evidence of this systematic review revealed that the use of digital technology impacts girls more negatively than boys, especially as a consequence of the use of social media (*p* < 0.005). These findings are consistent with previous research, which found similar trends (Montag and Elhai, 2020; Lehtimaki et al., 2021; Marciano et al., 2021). According to literature, female youth use digital technologies, and in particular the Internet, to seek feedback es from others (Valkenburg et al., 2005), and as the tone of feedback has been linked to self-evaluations (Valkenburg et al., 2006), it is possible that they perceive some of the feedback to be negative, which has subsequently influenced their adjustment (Neira and Barber, 2014).

Moreover, the results of this study indicate that adolescents experienced adverse mental health effects, including feelings of social isolation, depression, anxiety, and increases in maladaptive behavior as a result of increased digital technology usage during the COVID-19 pandemic (Limone and Toto, 2021). However, to the best of our knowledge, there is currently no research related to these variables or available in population groups similar to this paper's. Thus, these results cannot be compared nor can inferences be extrapolated. As such, future research is encouraged to investigate this further.

This study significantly contributes to the psychological education of technology in a variety of ways. Initially, the study found little statistically significant or negative connotation amongst digital-screen engagement and wellbeing in adolescents. Instead, largely negative relations were found in studies employing both self-reports of technology use and wellbeing measures, which could be a result of traditional method variance or discrepancies in such large-scale questionnaire data. This is in line with results from previous research presenting associations between digital-technology use and psychological or emotional outcomes in adolescents (Twenge et al., 2018a,b; Orben and Przybylski, 2019). Consequently, these collective results may infer that there is a minor significant negative association between technology use and psychological or emotional outcomes, which may be microscopic when compared with other activities in an adolescent's life (Orben and Przybylski, 2019).

Third, this study was also one of the first to examine whether digital-screen engagement before bedtime is especially detrimental to adolescent psychological wellbeing. Public opinion seems to be that using digital screens immediately before bed may be more harmful for teens than screen time spread throughout the day. Our exploratory and confirmatory analyses provided very mixed effects: some were negative, while others were positive or inconclusive. Our study, therefore, suggests that technology use before bedtime might not be inherently harmful to psychological wellbeing, even though this is a well-worn idea both in the media and in public debates.

The studies included in this review also highlighted positive outcomes. For example, Neira and Barber (2014) found that males with a Social Network Site (SNS) profile reported higher self-esteem levels than did males without an SNS profile, supporting the hypothesis that SNS could be associated with a positive social self-concept. Also results from Sanders et al. (2019) support the less-is-better hypothesis–qualified showing that educational screen time was associated with positive educational outcomes. Educational screen time, therefore, appears beneficial, suggesting that the detrimental effects may be domain-specific. In addition to this, the findings from Jensen et al. (2019), showing that frequent texters are the least depressed, are consistent with the extant literatures on social connections both online and face to face (Seabrook et al., 2016). Daily text messaging has been found to be associated with less daily depression symptoms, as adolescents reported lower levels of depression on days when they were most connected to others online *via* text messaging (George et al., 2018).

The limited quantity of studies used in this review, of which most were non-randomized in design, is a limitation. Because of this, some studies were subjected to risk of bias due to methodological techniques used, as evidenced in scores from quality assessment tools. Nevertheless, the findings from this systematic review are advantageous in encouraging further research exploring the impact of digital technology use during the COVID-19 pandemic among adolescence and, perhaps, cohorts younger than 14 years old.

The psychological and emotional wellbeing of adolescents following the impact of digital technology use is an imperious global and public health alarm, particularly as epidemiology advises of rising technology use across geographies. The results from this systematic review suggests robust connotations between the frequency of digital technology use and negative psychological and emotional behaviors and outcomes among adolescents aged 14–18 years old, which are consistent with previous studies in this field. Given that technology has become entrenched within the livelihood of adolescents, it is paramount to understand its influence on health and wellbeing. Although digital technology and media screen use has constructive benefits, such as enriching a learning environment, mounting data also suggests that misuse and overuse has adversative effects on a wide range of cognitive and emotional/behavioral complications. Childhood and adolescence are perilous and critical opportunities for development and throughout which youth tend to be predominantly vulnerable to the undesirable psychological effects of digital technology screen media usage. Therefore, rendering more research in this area indispensable.

More specifically, while research studies have extensively investigated the effects of screen media overuse on sleep disturbances, there continues to be conflicting data with regard to internalizing mechanisms, such as psychological and emotional wellbeing, as well as potential bidirectional relationships shared with the adverse outcomes discussed in this review. The comprehension of its impacts and its associated mechanisms are vital to producing screen time recommendations and guidelines and developing effective prevention/intervention strategies to alleviate screen media overuse and its adversative outcomes in children and adolescents.

Finally, it is vital for public health practitioners and policy makers to propose targeted public health interventions that are synergistic in their action, comprising several variables linked to technology mediums, recommended usage times, and social media app guidance for guardians of children this age. The small quantity of studies encompassed in this systematic review should not be discounted, but rather used as a foundation for further investigation on the relationship between digital technology use and the psychological and emotional outcomes in children.

The findings of this review should also be interpreted in light of the limitations of this work. First, English-language literature and studies published between 2017 and 2020 have been assessed and may, therefore, this study has overlooked significant findings reported in other languages or published in other years. Second, although the author aimed to conduct an exhaustive search, a relevant search term may have been omitted and consequently relevant studies may have not been retrieved. Third, although there has been the attempted to screen the retrieved studies thoroughly, it is possible that some salient studies were overlooked.

## Conclusion

In summary, this systematic review concludes that there is strong evidence of a relationship between each independent variable (digital technology use prior to and during the COVID-19 pandemic) and negative psychological and emotional outcomes among adolescents aged 14–18 years old. This systematic review also documents notable insignificant associations between general health of adolescents despite rising rates of technology usage (Aloufi et al., 2022; Guldager et al., 2022). Further studies are encouraged to assess the inconsistencies among these results. As the evidence in this review is compelling, it is important to emphasize the detrimental outcomes that amplified technology use has on the development of adolescents during this vulnerable phase of life (Borcoman and Sorea, 2022; Sokugawa, 2022). Therefore, it is important that further research on a larger scale, continues to assess any present synergistic relationships among these variables, in order to better advice public health initiatives dealing with technology use by children.

## Data Availability Statement

The original contributions presented in the study are included in the article/supplementary material, further inquiries can be directed to the corresponding author.

## Author Contributions

PL: introduction and conclusion. GT: methodology, results, and discussion. Both authors contributed to the article and approved the submitted version.

## Conflict of Interest

The authors declare that the research was conducted in the absence of any commercial or financial relationships that could be construed as a potential conflict of interest.

## Publisher's Note

All claims expressed in this article are solely those of the authors and do not necessarily represent those of their affiliated organizations, or those of the publisher, the editors and the reviewers. Any product that may be evaluated in this article, or claim that may be made by its manufacturer, is not guaranteed or endorsed by the publisher.
